# Screen Time Matters: Exploring the Behavioral Effects of Devices on Saudi Children

**DOI:** 10.3390/ijerph22050741

**Published:** 2025-05-08

**Authors:** Faisal O. AlQurashi, Feeda S. Almensif, Fatimah H. Alkhabbaz, Karrar Y. Alkhawahir, Dana Abalkhail

**Affiliations:** 1 Department of Pediatrics, College of Medicine, King Fahd Hospital of the University, Imam Abdulrahman Bin Faisal University, Dammam 34224, Saudi Arabia; 2 College of Medicine, Imam Abdulrahman Bin Faisal University, Dammam 34224, Saudi Arabia

**Keywords:** ADHD, neurodevelopmental disorder, screen time, sociodemographic factors, SNAP-IV scale, children, physical activity, parental education, Saudi Arabia

## Abstract

Attention deficit hyperactivity disorder (ADHD) is a neurodevelopmental disorder characterized by inappropriate levels of inattention, hyperactivity, or impulsivity. Recent concerns suggest a potential link between increased screen time and the manifestation of ADHD symptoms. This study examined the relationship between screen time and ADHD-related symptoms in neurotypical children aged 3–18 years in the Eastern Province of Saudi Arabia. Data were collected through an online questionnaire completed by caregivers, focusing on screen time habits, ADHD-related symptoms (measured using the SNAP-IV scale), and potential confounders. A total of 324 children participated, with a slight male predominance (52.2%) and a median age of 9.07 years. Most of the children were Saudi nationals (97.5%) and resided in urban areas (70.7%). Using screens for more than 5 h was associated with higher total SNAP-IV scores. Multivariate analysis revealed that unrestricted screen time, related disorders, and lower maternal education were strongly associated with higher SNAP-IV scores. This study revealed a notable association between screen time characteristics, sociodemographic factors, and ADHD-related symptom severity in children in the Eastern Province of Saudi Arabia.

## 1. Introduction

Attention deficit hyperactivity disorder (ADHD) is a neurodevelopmental disorder characterized by inappropriate levels of inattention, hyperactivity, or impulsivity [1]. According to the *Diagnostic and Statistical Manual of Mental Disorders, Fifth Edition* (DSM-5), ADHD can be categorized into three subtypes: predominantly inattentive, predominantly hyperactive/impulsive, and a combined type [2]. It significantly affects all aspects of a child’s life, including academic performance, social skills, self-esteem, and family relationships [3]. The global prevalence of ADHD in children and adolescents is estimated to be 8%, with 10% of males and 5% of females diagnosed with ADHD [4]. In Saudi Arabia, the estimated prevalence of ADHD is 8%, which is similar to the global prevalence. However, it is higher among males, by 3–5%, than among females [5].

Screen time is defined as the amount of time spent using electronic or digital media devices, such as televisions, smartphones, tablets, or computers [6]. According to the Saudi Arabian Ministry of Health guidelines, no screen time is allowed for newborns and toddlers under 18 months of age, while children between the ages of 18 and 24 months are allowed a few minutes with parents present. Children between 2 and 5 years of age should limit their screen time to less than 1 h per day, and children between the ages of 5 and 18 years should have no more than 2 h of sedentary recreational screen time per day [7]. These guidelines were adapted from the 2016 American Academy of Pediatrics Guidelines on Media Use for Children. However, the use of smart devices has increased in work, studies, and daily life, resulting in children being exposed to electronic devices at a younger age and for longer periods than recommended [8].

Excessive screen time during childhood can negatively affect development and health [6]. Regarding screen time duration, in 2019 a systematic review of reviews that included 13 international studies found strong evidence linking more screen time in children/adolescents to increased obesity, depressive symptoms, poorer diet, and reduced quality of life. Weak associations were observed with behavioral issues, anxiety, lower fitness, and adverse cognitive/educational outcomes, while no significant links emerged for eating disorders, asthma, or cardiovascular risk factors [9]. A recent Filipino study by Dy AB et al. found that there is minimal negative impact on development if screen time is limited to less than 2 h; however, there may be poorer language development if it exceeds this limit. Additionally, children tended to use screen media more often when they were alone. However, this study only targeted children aged between 24 and 36 months; moreover, it did not investigate the effect of screen exposure on the manifestations of ADHD symptoms [10]. Previous studies have suggested an association between screen time and manifestation of ADHD symptoms. Regarding screen type and frequency, a meta-analysis of 45 studies that examined the relationship between screen media use (TV and video games) and ADHD-related behaviors (attention problems, hyperactivity, and impulsivity) in children and adolescents found a small but significant positive association between media use and ADHD-related behaviors, with stronger links to attention problems and impulsivity than hyperactivity [11].

A recent longitudinal study in the US by Hill et al., which analyzed 82 toddlers with a high familial risk of autism/ADHD, found that children later diagnosed with autism (15/82) or exhibiting ADHD concerns (17/82) had significantly more screen time at 18 months compared to their neurotypical peers. Greater early screen exposure predicted increased autism/ADHD symptoms and lower developmental achievement at ages 3–5, with receptive/expressive language deficits twice as likely in high-screen-time toddlers [12]. Another recent Indian study found a positive correlation between ADHD severity, parental stress levels, and increased screen time in children. However, the sample size was small, with only 56 participants, and the study focused on children aged between 2.5 and 6 years who had already been diagnosed with ADHD [13]. In contrast, a cross-sectional study carried out in China with 2452 participants found that there was no statistically significant association between ADHD symptoms and screen time after excluding overweight, obese, and multi-child families [14]. In summary, various international reports have highlighted the negative effect of high screen time on child development and behavior. After reviewing the literature, there are no existing Saudi studies on the effect of media use on children’s behaviors. The only relevant Saudi paper was about the effect of technology use on children’s physical activity [15].

Due to the lack of published research regarding the effect of media use on children’s behaviors in Saudi Arabia, our study aimed to provide insight into the effects of screen time on ADHD-related symptoms in neurotypical children aged between 3 and 18 years of both sexes. Additionally, we investigated the relationships between the type, duration, and frequency of screen activity and ADHD symptoms and compared them between the sexes.

## 2. Materials and Methods

This cross-sectional study was conducted from January 2024 to April 2024 to assess the relationship between screen time and the manifestation of ADHD-related symptoms in neurotypical children aged between 3 and 18 years. Data were collected using an online questionnaire completed by the children’s caregivers. Caregivers were defined as parents or individuals who had regular contact with the children and were familiar with their daily activities and behaviors. The participants were required to be able to read and understand either Arabic or English. The questionnaire was distributed through social media to residents of the Eastern Province of Saudi Arabia. Children diagnosed with any neurodevelopmental disorders (including a previous diagnosis of ADHD), neurological diseases, or psychiatric disorders were excluded. These included ADHD, intellectual disabilities, developmental delays, autism spectrum disorder, obsessive–compulsive disorder, developmental coordination disorder, cerebral palsy, learning disorders, genetic disorders, thyroid disorders, sensory impairments, epilepsy, mood disorders, and anxiety disorders.

Before data collection, we conducted a pilot study to assess the feasibility, reliability, and validity of the questionnaire. It included a small group of caregivers with children who resembled the target population. Face validation of the questionnaire was carried out by two independent Developmental–Behavioral Pediatricians. We carefully reviewed the participants’ feedback to refine and enhance the questionnaire. Prior to commencement, ethical approval was obtained from the Institutional Review Board (IRB) of Imam Abdulrahman Bin Faisal University (IRB-2024-01-041). The first section of the questionnaire provided brief information on the study, its objectives, and the committee conducting it. This was followed by a consent statement for participants to either agree to participate or withdraw from the study. No identifiable data were requested, and the confidentiality and privacy of the participants’ information were strictly maintained.

The questionnaire comprised four sections. The first section sought demographic data and information about the child’s medical and psychiatric histories. The next section focused on assessing the child’s screen time. The caregivers were asked to complete the survey according to their child’s habits within the past 3 months. It included questions on the number of days the child used a screen, the duration of use on weekdays and weekends in hours, the types of screens used, and whether the child required permission before using the screens. The third section was the Swanson, Nolan, and Pelham Rating Scale (SNAP-IV), which has been shown to have excellent reliability and validity [16]. SNAP-IV is a rating scale that is widely used by parents and teachers. It measures the core symptoms of attention deficit hyperactivity disorder (ADHD) in children up to the age of 18 years. While SNAP-IV scores have high sensitivity, they also have low specificity compared with clinician diagnoses. Furthermore, this tool is valuable as a valid outcome measure in randomized controlled trials and clinical settings, functioning best as a screening tool instead of a diagnostic tool for ADHD [17]. It contains 26 items, 18 of which align with the DSM-5 ADHD diagnostic criteria intended to differentiate between various presentations of ADHD, while the remaining 8 items highlight oppositional defiant disorder symptoms. The first nine items focus on inattention, while the last nine focus on hyperactivity–impulsivity. Each item has four response options that reflect the child’s behavior over the past 3 months. A score higher than the standard indicates that the child may be at risk of ADHD. For inattention, a score of 16 or higher indicates risk, while for hyperactivity/impulsivity, a score of 14 or higher indicates risk [18]. The final section of the questionnaire addressed potentially confounding parental variables such as having multiple children in the family; socioeconomic status; parental age, education, and jobs; and parental screen usage. Screens were defined as any surface on which an image appears in an electronic display. This included televisions, mobile devices, smart devices (tablets and computers), and game consoles.

### 2.1. Statistical Analysis

All data were analyzed using the Statistical Package for the Social Sciences version 26.0 (IBM Corp., Armonk, NY, USA). The Shapiro–Wilk test was used to assess data normality. Medians and interquartile ranges (IQRs) were used to present continuous numerical data, whereas frequencies and percentages were used to present nominal data. The Mann–Whitney U and Kruskal–Wallis tests were used to correlate the sociodemographic data with SNAP-IV score subsets, with statistical significance set at a *p*-value of less than 0.05. To evaluate the effect of every indicator on the SNAP-IV outcome variable, multivariate analysis was performed using a linear regression model for the variables that were found to be significant in the correlation tests.

### 2.2. Results

The questionnaire initially recruited a total of 359 subjects. However, 27 subjects were excluded due to the presence of a neurodevelopmental disorder (including a previous diagnosis of ADHD), a neurological disease, or a psychiatric disorder, and 8 were excluded due to exceeding the assigned age limit of the study. This study included 324 children, with a slight male predominance (52.2% boys and 47.8% girls). Most of the participants were Saudi nationals (97.5%), and the median age was 9.07 years. Moreover, most of the participants were in primary school (47.2%), and 70.7% resided in urban areas. Regarding physical activity, 68.5% of the children were involved in physical activity for over 2 h/day. Furthermore, 51.5% of the children required parental permission to use screens, which were used daily by 77.2% of them. On weekdays, 29.0% used screens for over 5 h, which increased to 38.0% on weekends (Table 1).

Table 2 shows that the fathers had a median age of 44.93 years. Nearly half of them had a bachelor’s degree (49.1%), while 23.1% had a high school education or lower. The fathers were primarily employed in the private sector as full-time workers (38.9%), with significant representation in full-time government roles (36.4%). Additionally, most of the fathers lived in urban areas (70.7%), and the household incomes predominantly fell between SAR 11,000 and SAR 20,000 (37.3%). The mothers had a slightly lower median age of 39.57 years. Most of the mothers had a bachelor’s degree (51.2%). The majority of the mothers were homemakers (59.9%), and like the fathers were Saudi nationals residing in urban areas. The most prominent type of screen use by the children was mobile devices (67.3%). In contrast, social media usage (80.5%) was found to be the most common type of screen use by the parents (Table 3).

**Figure 1:**
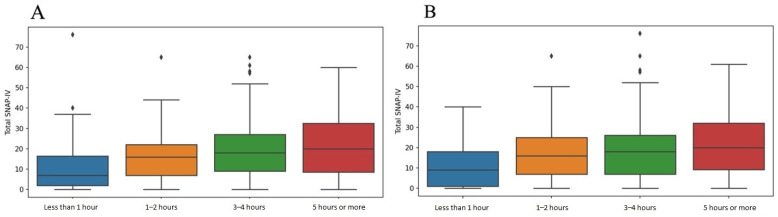
Boxplots summarizing the relationships between screen time and total SNAP-IV scores on (**A**) weekends and (**B**) weekdays.

Table 4 presents the details of the three SNAP-IV score subsets. The median values of the inattention subsets were 6.0, 5.0, and 6.0 for the opposition/defiance subset. The median value of the total SNAP-IV score was found to be 17.0. Figure 1 shows that higher total SNAP-IV scores were related to extended screen time on weekends, highlighting an increased possibility of ADHD-related symptoms. Similarly, higher total SNAP-IV scores were associated with longer screen usage on weekdays.

**Figure 2:**
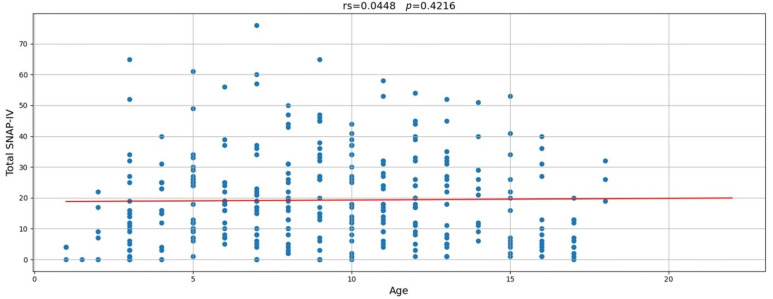
Correlation between age and total SNAP-IV score.

Figure 2 shows that there was no significant correlation between age and the total SNAP-IV score, indicating consistency in the possibility of ADHD-related symptoms across different ages. However, when age was categorized into four subgroups (3–5, 6–11, 12–14, and 15–18 years), the categorical assignment showed a significant association with elevated total SNAP-IV scores, with children between 6 and 11 years displaying higher scores than the other age groups (*p* = 0.005). Other sociodemographic factors were significantly associated with higher total SNAP-IV scores (Table 5). For instance, boys had significantly higher total SNAP-IV scores than girls (*p* = 0.020), and children with related disorders had significantly higher total SNAP-IV scores (*p* = 0.015). Additionally, maternal education showed significant results, where children of mothers with lower education levels had higher total SNAP-IV scores.

Furthermore, the screen time characteristics of the children and their parents were significantly correlated with the total SNAP-IV scores and their subsets (Table 6). For instance, children whose parents spent less than 2 h on screens showed lower inattention scores (*p* = 0.036), regardless of the child’s age. Additionally, children requiring parental permission for screen time showed lower total SNAP-IV (*p* = 0.007) and inattention scores (*p* = 0.002) than those without such limitations. On weekdays and weekends, children with 1–2 h of screen time had significantly lower total SNAP-IV, inattention, and hyperactivity/impulsivity scores than those with 5 h or more (*p* = 0.001).

The results of a multiple linear regression analysis of the factors associated with higher total SNAP-IV scores are shown in Table 7. This regression analysis showed that related disorders and unrestricted screen time were associated with higher total SNAP-IV scores (*p* = 0.029 and *p* < 0.001, respectively). Furthermore, increased weekday screen use (>5 h) added 8.91 points to the total SNAP-IV score (*p* < 0.001), while moderate weekend screen use (3–4 h) also elevated the score by 9.34 points (*p* < 0.001). Maternal education was another factor, with children of mothers with a diploma or a lower education level showing elevated scores (*p* < 0.001).

## 3. Discussion

This cross-sectional study examined the relationship between screen time and ADHD-related symptoms in children of both sexes in the Eastern Province of Saudi Arabia, spanning age groups from preschool to 18 years, including neurotypical children.

We found a strong correlation between more screen time and higher SNAP-IV scores, indicating ADHD-related symptoms (*p* < 0.001). This was consistent with the findings of Vaidyanathan et al., who identified an association between screen time and ADHD symptoms in preschool children [13], and Ahmer et al., who observed a significant relationship between increased screen time and exacerbated ADHD symptoms, including hyperactivity and inattention [19]. Meng et al. reported a similar association, proposing that excessive screen exposure may affect ADHD behaviors through neural mechanisms [20].

However, another study conducted by Zhou et al. concluded that no statistically significant correlation between screen time and ADHD symptoms was found after excluding multi-child families and children who were overweight or obese [14].

Electronic devices have become indispensable in children’s lives. Our study demonstrated that 250 (77.2%) of the 324 children used screens daily. The majority of the children were exposed to screens for 3–4 h on weekdays (119 children, 36.7%), with an increase to more than 5 h on weekends (123 children, 38.0%). This aligned with the findings of Almaqhawi and Albarqi, who reported similar trends of increasing screen time on weekends among children in the Eastern Province of Saudi Arabia [15]. These results support a global concern; McArthur et al. highlighted that excessive screen time is a common issue worldwide, with a significant percentage of children exceeding the recommended screen time limits [21].

The World Health Organization and Australian 24 h movement guidelines recommend no screen time for children younger than 2 years, screen exposure limited to 1 h for children aged between 2 and 5 years, and no more than 2 h of screen time for children aged between 5 and 17 years [7,22]. Despite these guidelines, our findings suggest that many children in the Eastern Province of Saudi Arabia exceed these recommended limits, contributing to growing concerns regarding the adverse effects of excessive screen exposure on the health of children. This observation could be due to the lack of parental control on media use, the availability of multiple screens in each household, or poor community education.

Interestingly, our study found a stronger association between weekday screen exposure and the severity of ADHD-related symptoms. This was particularly evident in children who were exposed to more screen time on weekdays; however, the variation between weekdays and weekends has not been widely discussed in other studies.

In our study, no significant association was found between age and total SNAP-IV scores, suggesting that ADHD-related symptoms may be consistent across different age groups in neurotypical children. However, an unadjusted analysis of categorized ages showed a significant association with elevated total SNAP-IV scores, with children aged 6 to 11 years displaying higher scores than the other age groups (*p* = 0.005). Unfortunately, this was not significant after adjusting the data for linear regression.

Sociodemographic factors, including sex, were significantly associated with ADHD-related symptom severity in our study, with males showing notably higher total SNAP-IV scores than females (*p* = 0.020). This was consistent with the findings of Ayano et al., who highlighted that ADHD is more prevalent among boys, with boys being twice as likely to be diagnosed with this disorder than girls [4]. This sex difference may be attributed to the fact that boys are more likely to exhibit hyperactive/impulsive symptoms, whereas girls often present with inattentive symptoms, which can lead to underdiagnosis among girls. This aligns with the findings of Slobodin and Davidovitch, who suggested that sex differences in ADHD symptoms contribute to the discrepancy in diagnostic rates between boys and girls [23].

We also found that children whose parents limited their screen time to less than 2 h per day and required parental permission for screen use had significantly lower SNAP-IV scores, indicating fewer ADHD-related symptoms. This highlighted the importance of parental control over screen use, as previously reported by Zhou et al. and Dy et al., who both found that parental involvement in screen time regulation was associated with reduced ADHD symptoms in children [10,14]. These findings are consistent with those of Vaidyanathan et al., who suggested that children with more regulated screen time exhibit less pronounced ADHD behaviors, emphasizing the protective role of active parental supervision [13]. In contrast, our study found that children who spent 5 h or more on screens exhibited higher levels of inattention, hyperactivity, and impulsivity compared to those who spent less than 2 h, which highlighted the importance of parental and community education on following the media use guidelines for screen restriction. One other significant factor that may result in more severe ADHD-related symptoms among children is their parents’ daily screen use. Our study found that parents’ using screens for more than two hours a day was significantly associated with higher inattentive scores among children. This observation was also reported by Dy AB [10]. This could be because parental use of screens may disrupt parent–child interactions by reducing parental responsiveness to children’s attention-seeking behaviors, potentially impairing the parent–child relationship. Additionally, higher parental screen exposure may result in increased screen exposure for children.

Furthermore, our research suggests that socioeconomic factors, particularly maternal education, are associated with more severe ADHD-related symptoms. This supports the findings of Arrirak et al., who observed that family income and maternal education significantly influenced the prevalence and management of ADHD symptoms in children in Yasothon, Thailand [18]. These results align with global studies that emphasize the role of socioeconomic status in shaping children’s health outcomes and access to ADHD management strategies [20].

Interestingly, no significant correlations were found between the number of children in a family and screen time or ADHD-related symptoms (*p* = 0.352). In contrast, Zhou et al. found a significant association between screen time and ADHD symptoms in families with multiple children, potentially because parents spend more time caring for younger children, which may inadvertently increase screen time for older children [14]. This variation underscores the complexity of family dynamics in influencing children’s screen time and their potential effects on attention, impulse control, and hyperactivity. Adolescent media consumption has increased over the past decade, driven by the widespread adoption of mobile phones among teenagers. Currently, approximately 95% of adolescents own a smartphone, providing continuous access to the Internet, streaming services, and interactive applications. Notably, nearly half of teenagers report being “constantly connected” to the Internet [24].

By comparing our findings with the existing literature, it became clear that our study reaffirmed the roles of screen time and parental supervision in shaping ADHD-related symptoms, with particular emphasis on the impact of screen habits on weekdays. However, the lack of age-related differences and associations with physical activity or sleep in our sample highlights the need for further investigation of these variables in diverse populations.

This study has several strengths. First, to the best of our knowledge, this is the first study in our region to explore the relationship between screen time and ADHD-related symptom severity across a broad age range (3–18 years). Second, a pilot study was conducted before data collection to assess the feasibility, reliability, and validity of the questionnaire. Feedback from caregivers and an experienced faculty member helped improve the clarity and the suitability of the study population. Third, this study adhered to strict ethical standards by obtaining approval from the IRB before commencement. The participants were informed of the study’s objectives, and a consent statement ensured their voluntary participation and right to withdraw at any time. Fourth, strict confidentiality and privacy were maintained, and no identifiable data were collected from the participants. Fifth, use of the SNAP-IV, a well-established and trusted tool for assessing ADHD-related symptoms, ensured accurate measurements and allowed for consistent comparisons with other research in this field.

However, this study also had a few limitations. First, it relied on self-reported data, with information collected through caregiver reports, which may be subject to recall or social desirability biases. In addition, although this study targeted neurotypical children, we cannot rule out the possibility of undiagnosed ADHD among the included cohort, as this would require medical evaluations. Second, this study did not consider the screen time content, which could influence a child’s behavior and ADHD-related symptoms. Third, this study did not incorporate an assessment of the caregivers’ perceptions regarding the impact of screen use on their children’s behavior, nor did it explore the caregivers’ views on whether the screen time habits require modification. Fourth, this study did not assess the time parents spent with their children or its correlation with the manifestations of ADHD symptoms. Fifth, this study used a unidirectional assessment tool (SNAP-IV) that focuses on assessing deficits and/or symptom severity. However, using a more comprehensive assessment tool like The Strengths and Weaknesses of Attention-Deficit/Hyperactivity Disorder Symptoms and Normal Behavior Scale (SWAN) may provide a more balanced assessment since it assesses these dimensions as a continuum of behaviors ranging from strengths to difficulties. Finally, this study mainly tested the effect of screen time on Saudi national children, which may limit the generalizability of the findings to other ethnic groups.

## 4. Conclusions

This study revealed notable associations between screen time characteristics, in addition to other environmental factors such as sociodemographic factors, individual factors, and familial screen time patterns, and ADHD-related symptom severity in children in the Eastern Province of Saudi Arabia. Further prospective studies are needed to investigate these relationships and confirm their generalizability. Our study may serve as a valuable tool that allows healthcare providers to develop tailored management approaches for children with ADHD-related symptoms and unrecommended screen exposure. By identifying key factors, such as screen time habits, parental behaviors, and sociodemographic influences, our findings may guide personalized interventions that account for different social backgrounds. Future research should focus on analyzing the specific content of screen time to better understand its influence on ADHD-related symptoms, moving beyond the current emphasis on screen time duration alone.

## Figures and Tables

**Table 1:** The sociodemographic characteristics of the children involved in this study.

Study Variables	Total Patients
Sex	
Male	169 (52.2%)
Female	155 (47.8%)
Age, years (median ± IQR)	9.07 ± 6.00
Nationality	
Saudi	316 (97.5%)
Non-Saudi	8 (2.5%)
Height, cm (median ± IQR)	130.00 ± 46.75
Weight, kg (median ± IQR)	30.00 ± 24.50
Related Disorders	
Yes	27 (8.3%)
No	297 (91.7%)
Age, years	
3–5	88 (27.2%)
6–11	153 (47.2%)
12–14	46 (14.2%)
15–18	37 (11.4%)
The number of students in the class	
1 to 10	39 (12.0%)
11 to 20	62 (19.1%)
21 to 30	108 (33.3%)
31 to 40	96 (29.6%)
41 or more	19 (5.9%)
Premature birth (before 37 weeks of gestation)	
Yes	27 (8.3%)
No	297 (91.7%)
Duration of daily sleep, hours	
<4	2 (0.6%)
5–9	208 (64.2%)
10–14	113 (34.9%)
>14	1 (0.3%)
Duration of physical activity, hours	
<2	102 (31.5%)
≥2	222 (68.5%)
Needs permission for screen time	
Yes	167 (51.5%)
No	157 (48.5%)
Use of screens	
Weekdays	41 (12.7%)
Weekend	33 (10.2%)
All days	250 (77.2%)
Screen time on weekdays, hours	
<1	27 (8.3%)
1–2	84 (25.9%)
3–4	119 (36.7%)
≥5	94 (29.0%)
Screen time on weekend, hours	
<1	31 (9.6%)
1–2	61 (18.8%)
3–4	109 (33.6%)
≥5	123 (38.0%)

IQR, interquartile range.

**Table 2:** The sociodemographic characteristics of the parents involved in this study.

Study Variables	Total Patients
Number of children in family	
1	69 (21.3%)
≥2	255 (78.7%)
Paternal age, years (median ± IQR)	44.93 ± 11.00
Paternal nationality	
Saudi	313 (96.6%)
Non-Saudi	11 (3.4%)
Paternal education	
Uneducated	1 (0.3%)
High school and below	75 (23.1%)
Diploma	72 (22.2%)
Bachelor’s	159 (49.1%)
PhD	17 (5.2%)
Paternal occupation	
Unemployed	12 (3.7%)
Full-time private	126 (38.9%)
Part-time private	19 (5.9%)
Full-time governmental	118 (36.4%)
Part-time governmental	16 (4.9%)
Retired	33 (10.2%)
Maternal age, years (median ± IQR)	39.57 ± 11.00
Maternal nationality	
Saudi	314 (96.9%)
Non-Saudi	10 (3.1%)
Maternal education	
Uneducated	1 (0.3%)
High school and below	104 (32.1%)
Diploma	39 (12.0%)
Bachelor’s	166 (51.2%)
PhD	14 (4.3%)
Maternal occupation	
Stay-at-home mother	194 (59.9%)
Full-time private	27 (8.3%)
Part-time private	17 (5.2%)
Full-time governmental	70 (21.6%)
Part-time governmental	7 (2.2%)
Retired	9 (2.8%)
Monthly household income, SAR	
≤2000	6 (1.9%)
3000–5000	40 (12.3%)
6000–10,000	91 (28.1%)
11,000–20,000	121 (37.3%)
≥21,000	66 (20.4%)
Duration of parents staying at jobs each day, hours	
>7	144 (44.4%)
≤7	180 (55.6%)
Type of residence	
Urban	229 (70.7%)
Rural	95 (29.3%)
Parent’s screen time, hours	
≥2	224 (69.1%)
<2	100 (30.9%)

IQR, interquartile range; SAR, Saudi riyal.

**Table 3:** Types of screen use among parents and their children.

Type of Screen Use	Total Patients
Children	
Television	187 (57.7%)
Mobile devices	218 (67.3%)
Computers/tablets	128 (39.5%)
Game consoles	63 (19.4%)
Parents	
Television	200 (61.7%)
Video calls	45 (13.9%)
Social media	261 (80.5%)
Web browsing	138 (42.6%)
Gaming	38 (11.7%)
Others	3 (0.9%)

**Table 4:** Frequencies and medians of SNAP-IV items for each subset.

SNAP-IV Items	0	1	2	3
Total Inattention Subset (median ± IQR)	6.0 ± 7.0			
Often fails to give close attention to details or makes careless mistakes in schoolwork or tasks	130 (40.1%)	147 (45.4%)	34 (10.5%)	13 (4.0%)
Often has difficulty sustaining attention in tasks or play activities	186 (57.4%)	96 (29.6%)	33 (10.2%)	9 (2.8%)
Often does not seem to listen when spoken to directly	146 (45.1%)	125 (38.6%)	38 (11.7%)	15 (4.6%)
Often does not follow through on instructions and fails to finish schoolwork, chores, or duties	147 (45.4%)	129 (39.8%)	38 (11.7%)	10 (3.1%)
Often has difficulty organizing tasks and activities	152 (46.9%)	121 (37.3%)	37 (11.4%)	14 (4.3%)
Often avoids, dislikes, or reluctantly engages in tasks requiring sustained mental effort	151 (46.6%)	113 (34.9%)	40 (12.3%)	20 (6.2%)
Often loses things necessary for activities (e.g., toys, school assignments, pencils, or books)	151 (46.6%)	126 (38.9%)	34 (10.5%)	13 (4.0%)
Often is distracted by extraneous stimuli	118 (36.4%)	133 (41.0%)	50 (15.4%)	23 (7.1%)
Often is forgetful in daily activities	166 (51.2%)	114 (35.2%)	33 (10.2%)	11 (3.4%)
Total Hyperactivity/Impulsivity Subset (median ± IQR)	5.0 ± 8.0			
Often fidgets with hands or feet or squirms in seat	159 (49.1%)	103 (31.8%)	37 (11.4%)	25 (7.7%)
Often leaves seat in classroom or in other situations in which remaining seated is expected	199 (61.4%)	95 (29.3%)	24 (7.4%)	6 (1.9%)
Often runs about or climbs excessively in situations in which it is inappropriate	190 (58.6%)	96 (29.6%)	28 (8.6%)	10 (3.1%)
Often has difficulty playing or engaging in leisure activities quietly	199 (61.4%)	84 (25.9%)	29 (9.0%)	12 (3.7%)
Often is “on the go” or often acts as if “driven” by a motor	221 (68.2%)	63 (19.4%)	33 (10.2%)	7 (2.2%)
Often talks excessively	169 (52.2%)	88 (27.2%)	48 (14.8%)	19 (5.9%)
Often blurts out answers before questions have been completed	145 (44.8%)	107 (33.0%)	51 (15.7%)	21 (6.5%)
Often has difficulty awaiting turn	172 (53.1%)	112 (34.6%)	29 (9.0%)	11 (3.4%)
Often interrupts or intrudes on others (e.g., butts into conversations/games)	156 (48.1%)	116 (35.8%)	36 (11.1%)	16 (4.9%)
Total Opposition/Defiance Subset (median ± IQR)	6.0 ± 7.25			
Often loses temper	122 (37.7%)	121 (37.3%)	51 (15.7%)	30 (9.3%)
Often argues with adults	126 (38.9%)	117 (36.1%)	49 (15.1%)	32 (9.9%)
Often actively defies or refuses adult requests or rules	112 (34.6%)	129 (39.8%)	52 (16.0%)	31 (9.6%)
Often deliberately does things that annoy other people	173 (53.4%)	102 (31.5%)	32 (9.9%)	17 (5.2%)
Often blames others for his or her mistakes or misbehaviour	157 (48.5%)	121 (37.3%)	26 (8.0%)	20 (6.2%)
Often touchy or easily annoyed by others	91 (28.1%)	138 (42.6%)	60 (18.5%)	35 (10.8%)
Often is angry and resentful	131 (40.4%)	129 (39.8%)	45 (13.9%)	19 (5.9%)
Often is spiteful or vindictive	215 (66.4%)	71 (21.9%)	27 (8.3%)	11 (3.4%)
Total SNAP-IV score (median ± IQR)	17.0 ± 20.0			

Note: responses were categorized into 0, 1, 2, and 3, representing “Not at all”, “A little bit”, “Quite a bit”, and “Very much”, respectively. IQR, interquartile range.

**Table 5:** Correlations between sociodemographic factors and total SNAP-IV scores.

Factor	Total SNAP-IV Score/Correlation	Z/H-Test *p*-Value
Sex		
Male	21.05 ± 15.26	−2.325
Female	17.27 ± 13.99	0.020 *
Age categories, years		
3–5	16.70 ± 13.55	12.694
6–11	22.18 ± 15.55	0.005 *
12–14	17.98 ± 13.42	
15–18	14.70 ± 13.44	
Nationality		
Saudi	19.36 ± 14.89	−0.749
Non-Saudi	14.38 ± 8.32	0.454
Height	−0.03	0.546
Weight	0.02	0.675
Related Disorders		
Yes	25.37 ± 14.25	−2.433
No	18.68 ± 14.71	0.015 *
The number of students in the class		
1–10	18.72 ± 15.87	4.882
11–20	18.16 ± 13.09	0.300
21–30	21.16 ± 16.19	
31–40	18.97 ± 13.34	
≥41	14.32 ± 14.57	
Premature birth (before 37 weeks of gestation)		
Yes	18.26 ± 15.85	−0.539
No	19.33 ± 14.68	0.590
Duration of child’s daily sleep, hours		
<4	51.50 ± 24.50	5.882
5–9	19.48 ± 14.20	0.117
10–14	18.36 ± 14.96	
>14	4.00 ± 0.00	
Duration of child’s physical activity, hours		
<2	21.45 ± 15.27	−1.874
≥2	18.23 ± 14.45	0.061
Number of children in family		
1	18.30 ± 15.64	−0.930
≥2	19.49 ± 14.54	0.352
Paternal age, years	0.09	0.089
Paternal nationality		
Saudi	19.40 ± 14.92	−0.814
Non-Saudi	14.73 ± 9.08	0.416
Paternal education		
Uneducated	13.00 ± 0.00	3.660
High school and below	18.97 ± 16.42	0.454
Diploma	21.96 ± 15.10	
Bachelor’s	18.23 ± 13.64	
PhD	18.71 ± 15.15	
Paternal occupation		
Unemployed	22.33 ± 16.96	2.552
Full-time private	19.75 ± 15.20	0.769
Part-time private	22.37 ± 15.67	
Full-time governmental	18.66 ± 13.63	
Part-time governmental	15.94 ± 12.51	
Retired	18.03 ± 16.13	
Maternal age, years	0.08	0.135
Maternal nationality		
Saudi	19.08 ± 14.74	−0.991
Non-Saudi	24.20 ± 15.48	0.321
Maternal education		
Uneducated	45.00 ± 0.00	10.141
High school and below	22.23 ± 16.26	0.038 *
Diploma	20.31 ± 11.76	
Bachelor’s	17.05 ± 14.09	
PhD	18.14 ± 13.51	
Maternal occupation		
Stay-at-home mother	19.79 ± 15.78	8.364
Full-time private	25.67 ± 15.83	0.137
Part-time private	18.76 ± 11.39	
Full-time governmental	16.00 ± 11.63	
Part-time governmental	19.29 ± 10.69	
Retired	14.11 ± 11.17	
Monthly household income, SAR		
≤2000	24.83 ± 16.69	2.348
3000–5000	19.55 ± 15.12	0.672
6000–10,000	20.78 ± 15.30	
11,000–20,000	18.35 ± 14.14	
≥21,000	18.06 ± 14.53	
Duration of parents staying at jobs each day, hours		
>7	19.42 ± 15.00	−0.131
≤7	19.09 ± 14.61	0.896
Type of residence		
Urban	18.97 ± 15.02	−0.745
Rural	19.88 ± 14.19	0.456

Note: * *p*-value < 0.05; SAR, Saudi riyal.

**Table 6:** Correlations between SNAP-IV subsets and screen time characteristics.

Factor	Total SNAP-IV Score	Z/H-Test *p*-Value	Total Inattention Subset Score	Z/H-Test *p*-Value	Total Hyperactivity/Impulsivity Subset Score	Z/H-Test *p*-Value
Parent’s screen time, hours						
≥2	19.75 ± 14.84	−1.041	6.94 ± 5.50	−2.086	6.03 ± 5.58	−0.973
<2	18.11 ± 14.61	0.298	6.08 ± 6.28	0.036 *	5.24 ± 4.95	0.328
Child needs permission for screen time						
Yes	16.95 ± 13.34	−2.713	5.70 ± 5.27	−3.121	5.37 ± 5.03	−1.106
No	21.68 ± 15.82	0.007 *	7.71 ± 6.08	0.002 *	6.24 ± 5.74	0.266
Use of screens						
Weekdays	16.41 ± 12.81	1.837	5.44 ± 4.96	3.702	5.29 ± 4.59	0.221
Weekend	17.03 ± 11.85	0.399	5.36 ± 4.26	0.157	5.76 ± 4.83	0.895
All days	20.00 ± 15.34		7.05 ± 6.00		5.87 ± 5.59	
Screen time on weekdays, hours						
<1	11.37 ± 11.43	15.871	3.48 ± 4.70	23.243	3.26 ± 3.82	8.074
1–2	16.93 ± 12.78	0.001 *	5.70 ± 4.83	0.000 **	5.62 ± 5.15	0.045 *
3–4	20.04 ± 15.17		6.60 ± 5.59		6.34 ± 5.72	
≥5	22.55 ± 15.64		8.55 ± 6.36		5.96 ± 5.38	
Screen time on weekends, hours						
<1	13.19 ± 16.32	16.308	4.03 ± 5.98	26.412	4.06 ± 6.01	8.354
1–2	15.89 ± 12.09	0.001 **	5.05 ± 5.05	0.000 **	5.41 ± 4.77	0.039 *
3–4	19.83 ± 14.27		6.80 ± 5.33		6.13 ± 5.31	
≥5	21.90 ± 15.28		8.03 ± 5.98		6.11 ± 5.53	

Note: * *p*-value < 0.05; ** *p*-value < 0.001.

**Table 7:** A multivariate linear regression analysis of the factors associated with higher total SNAP-IV scores.

Variables	Adjusted Linear Regression		
	*p*-Value	Coef	95% Confidence Interval (Lower–Upper)
Female	0.666	−0.746	(−4.143–2.651)
Related disorders	0.029 *	7.206	(0.762–13.651)
Age category (reference = 6–11 years)			
3–5 years	0.201	2.778	(−1.492–7.048)
12–14 years	0.391	−2.251	(−7.402–2.901)
15–18 years	0.066	−5.464	(−11.296–0.367)
No need for permission for screen time	0.000 **	10.064	(6.322–13.805)
Screen time on weekdays (reference = 3–4 h)			
<1 h	0.801	−1.006	(−8.866–6.854)
1–2 h	0.115	3.794	(−0.935–8.523)
≥5 h	0.000 **	8.912	(4.291–13.533)
Screen time on weekend (reference = 5 h or more)			
<1 h	0.061	7.230	(−0.340–14.800)
1–2 h	0.006 *	7.672	(2.172–13.172)
3–4 h	0.000 **	9.340	(5.368–13.313)
Maternal education (reference = Bachelor’s)			
Uneducated	0.089	26.770	(−4.138–57.678)
High school and below	0.000 **	7.664	(3.794–11.533)
Diploma	0.022 *	6.408	(0.936–11.880)
PhD	0.136	6.496	(−2.052–15.044)

Note: * *p*-value < 0.05; ** *p*-value < 0.001.

## Data Availability

The original contributions presented in this study are included in this article. Further inquiries can be directed to the corresponding authors.
